# The Cultural Evolution of Human Communication Systems in Different Sized Populations: Usability Trumps Learnability

**DOI:** 10.1371/journal.pone.0071781

**Published:** 2013-08-15

**Authors:** Nicolas Fay, T. Mark Ellison

**Affiliations:** School of Psychology, University of Western Australia, Perth, Australia; University of Stirling, United Kingdom

## Abstract

This study examines the intergenerational transfer of human communication systems. It tests if human communication systems evolve to be easy to learn or easy to use (or both), and how population size affects learnability and usability. Using an experimental-semiotic task, we find that human communication systems evolve to be easier to use (production efficiency and reproduction fidelity), but harder to learn (identification accuracy) for a second generation of naïve participants. Thus, usability trumps learnability. In addition, the communication systems that evolve in larger populations exhibit distinct advantages over those that evolve in smaller populations: the learnability loss (from the Initial signs) is more muted and the usability benefits are more pronounced. The usability benefits for human communication systems that evolve in a small and large population is explained through guided variation reducing sign complexity. The enhanced performance of the communication systems that evolve in larger populations is explained by the operation of a content bias acting on the larger pool of competing signs. The content bias selects for information-efficient iconic signs that aid learnability and enhance usability.

## Introduction

Human communication systems such as language evolve, and socio-cultural processes play a crucial role [1], [2]. Laboratory experiments have proved to be particularly useful to the study of cultural phenomena that leave no physical trace (e.g., natural language) as they provide an opportunity to model pre-historical processes. By creating a situation where human participants must communicate to a partner without using their conventional language system, investigators are able to study the origin and evolution of simple communication systems among modern-day humans (for a review see [3], [4]). By isolating the psychological variables important to the evolution of human communication systems, the results of laboratory experiments complement the findings of naturalistic and computer simulation approaches.

In this paper we use an experimental approach to test if human communication systems culturally evolve to be easily acquired by later generations. More specifically, we ask if communication systems evolve to be easy to learn, or to be easy to use, or both. We also examine the effect of population size on the learnability and usability of the evolved communication systems. First we review artificial language learning studies that suggest that human communication systems primarily evolve to be learnable. Next we review experimental-semiotic studies that suggest that human communication systems primarily evolve to be usable (i.e., efficient to produce and faithfully reproduced). We discuss the effect of population size on the evolution of human communication systems before reporting the results of the present experiment.

### Artificial Language Learning Approaches to Studying Language Evolution

For a linguistic pattern to survive and propagate, people must be able to learn and use it. “Today’s language is the product of yesterday’s learners” in as much as language learners must acquire their local linguistic system if they are to communicate successfully with the other members of their community [5]. Experimental approaches to language evolution typically use an artificial language learning paradigm, where participants are trained on a grammatically novel miniature language that is provided by the experimenter. Sometime later participants recall the acquired system and experimenters study how the system changes as a consequence of the users’ cognitive biases (e.g., [6]–[8]).

To examine the cognitive biases that guide language evolution, researchers often use a linear transmission or diffusion chain design [9]. This design is frequently used in experimental studies of cultural evolution [10]–[13]. The first person in the chain is trained on a miniature artificial language that s/he must later recall. What they recall then serves as training data for the next person in the chain. Subsequent members of the chain follow this training then recall procedure. This approach was used to study the emergence of linguistic structure by [7]. In this study transmission chains were initiated with a sequence of random letter sequences that communicated a set of objects that varied with regard to shape, colour and movement. Iterated across generations the initially random holistic systems became increasingly structured, reflecting broad distinctions between the categories of objects. Thus, a cognitive bias for compositional structure became amplified through repeated intergenerational cultural transmission. The cumulative increase in language-like structure had the benefit of making the system more learnable for those who encountered the artificial language later in the transmission chain (reflected by less learning error). However, this came at a cost – the system became increasingly underspecified and ambiguous; the number of unique labels was systematically reduced and the number of objects associated with each label increased. Thus, what emerged was a system that was optimized for learning, but whose functional value in terms of communication was sacrificed due to an increase in homonymy. In other words, the pressure for learnability trumped the pressure for usability.

In this study non-functional language systems may have ‘evolved’ because there was no communication pressure; participants recalled the linguistic data without using it to communicate to another person. In the next section we discuss experimental-semiotic studies in which participants’ explicit goal is to create a sign system that can be used to communicate a range of meanings to their partner.

### Experimental-Semiotic Approaches to Studying Language Evolution

Experimental-semiotic approaches study the evolution of signs and systems of signs in the absence of pre-established linguistic conventions. Typically participants communicate in a novel modality, e.g., by drawing [14]–[18], through gesture [19] or movement [20] and the experimenters study how the sign system evolves across repeated interactions between the agents. This approach benefits from being relatively unconstrained; unlike artificial language learning paradigms, the initial state of the system and the signs used to communicate are not pre-specified by the experimenter.

**Figure 1:**
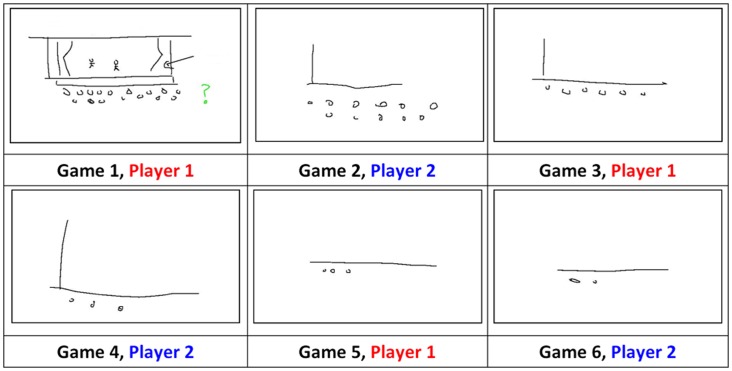
Sign refinement and alignment for the item *Theatre* across six games between a 2-Person group playing the Pictionary-like task (sampled from the corpus collected by [21]). Participants alternate drawing and identifying roles from game to game.

A key finding is that recurring social interactions cause initially iconic signs to evolve into more language-like symbolic signs [15], [18], [21]. In [15] participants communicated a series of recurring referents (e.g., *Art Gallery*, *Drama*, *Theater*) to a partner by drawing on a shared whiteboard. Like the game Pictionary, participants were not allowed to speak or use letters or numbers in their drawings. As participants repeatedly played the game the form of the sign used to communicate each referent changed; for example, at Game 1 the sign used to communicate *Theater* is a visually complex iconic drawing of a theater, including a stage, curtains, actors and an audience, whereas by game 6 it has evolved into a simple symbolic drawing, communicated by a line and two circles (see Figure 1). Sign refinement is only seen when participants interact with a partner. Repeated drawing without interaction did not lead to such simplification and abstraction (in fact, the drawings became more complex).

For those actively involved in the communication task, cumulative sign refinement led to a set of signs that became increasingly more efficient to execute. This optimization for efficient expression is consistent with guided variation (i.e., directed variant modification through individual use [22]). In the Figure 1 example interacting partners simplified the initial variant in a way that reduced their production effort. Although this production efficiency did not affect comprehension for participants who were actively involved in the communication task (their identification rates improved across repeated interactions), there was a learning cost for non-active participants; the referent of the symbolic signs was harder to identify compared to the earlier, more information rich iconic signs [15]. Thus, and in contrast to artificial language learning experiments, when a communication pressure is introduced usability trumps learnability.

### Population Size

Correlational studies show that population size is associated with linguistic complexity; larger populations have structurally simpler languages than smaller populations [23]. We propose that larger populations generate more linguistic variants that are subject to cultural selection, leading to the retention of linguistic rules that are easier to acquire.

**Figure 2:**
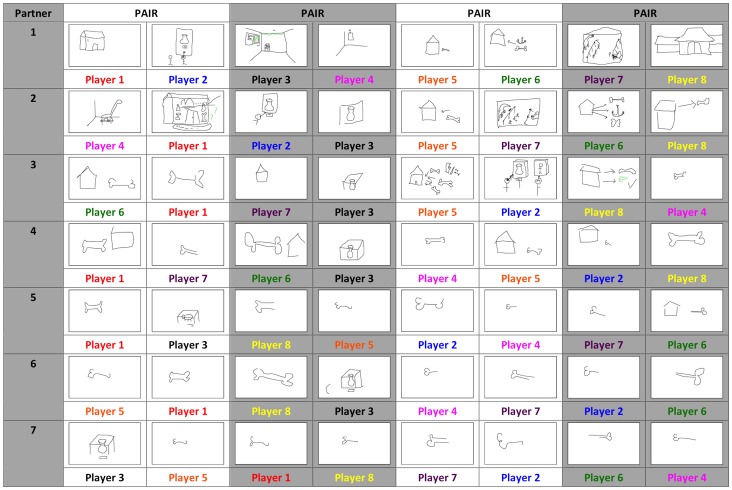
Sign refinement and alignment for the item *Museum* among members of an 8-Person group playing the Pictionary-like task (sampled from the corpus collected by [21]). Participants interact with each of the other members of their group (Partner 1–7). Drawings are sampled from Game 1 and Game 2 for each interacting pair (Game 1 drawings are presented on the left for each Pair). Shading is used to discriminate the different participant pairings.

The effect of population size was examined in an experimental-semiotic task that compared the sign systems that evolved in small 2-Person groups and larger 8-Person groups [21]. In the 8-Person groups participants played the Pictionary-like drawing game 6 times with their partner before switching partners and playing the games 6 times with a new partner. They continued in this way until they had interacted with each of the seven members of their community (i.e., participants played 42 games in total). Members of the 2-Person groups played the same number of games in total but always with the same partner. Figure 2 illustrates the cultural evolution of the signs used to communicate *Museum* in an 8-Person group. Initially (when participants play with their first partner, Partner 1) a variety of different iconic signs are used by participants: two people looking at an exhibit, a three-dimensional room with a painting mounted on the wall, a building that contains a dinosaur behind an enclosure and a building with a bone to the right of it. As players interact with the other members of their group, the building plus bone sign propagates until everyone is using a refined version of this sign (a schematic bone drawing) by the time they meet their final partner (Partner 7, with the exception of Player 3). A similar process is seen in natural language studies [24] and computer simulations [25], [26]. In both cases local pairwise interactions allow a representation to spread among a population of agents until the entire community aligns on the same communication system.

In the small and large groups initial sign complexity is dramatically reduced over the course of the experiment. Thus, in both conditions guided variation is evident (directed variant modification through individual use [22]). In both conditions participants aligned on a shared communication system; members of the 2- and 8-Person groups tended to use the same signs as the other members of their group to communicate the same referents. Where the two conditions differ is in the degree of sign variation; initially there is considerably more (but proportionate) variation in the larger populations. Given the preference of interacting pairs to use one sign to communicate one meaning, this creates greater competition among the signs in the larger populations. If sign selection is driven by a content bias (i.e., a particular sign is adopted because of its favorable content [22]) then, in a larger pool of competitors, we can expect the result of this winnowing to be better signs (e.g., signs that are easier to learn or use).

To examine this [27] presented these signs (Initial and Late, 2-Person and 8-Person signs) to a naïve second generation of participants who had to learn which sign went with which referent before being tested on a speeded recognition task. They found that the Late, more symbolic signs from each condition were harder to identify on first encounter compared to the Initial more iconic signs. Thus, the signs became *less* learnable (where learnability is operationalized as ease of comprehension). Interestingly, the extent to which learnability was sacrificed was more muted in the larger populations; naïve participants were better able to identify the referent of the Late signs that evolved in the larger groups compared to the smaller groups. Importantly, once learnt, the meaning of the signs that evolved in the larger populations were more rapidly accessed (compared to the Initial and Late signs from the 2-Person group). These differences between the Late evolved signs from the small and larger populations occurred despite the signs being of comparable visual complexity.

Despite their comparable visual complexity, the signs that evolved in the larger populations retained greater residual iconicity and this speeded sign processing and reduced the loss in sign learnability by naïve participants. The residual iconicity of the signs that evolved in the 8-Person groups is seen in Figure 2, where the simplified ‘bone’ sign used to communicate *Museum* is more iconic than the equally simple sign for *Theater* that evolved in a 2-Person group (Figure 1).

### The Present Study

For a communication system to survive and propagate in a population its users must be able to learn the relevant sign-meaning mappings and faithfully reproduce the signs. Thus, accurate comprehension and production are important evolutionary pressures for a functional communication system. The parity requirement has it that language is a compromise between ease of comprehension for the listener and ease of production for the speaker [28], [29]. Whereas speakers want to minimise their production effort, and therefore tend toward briefer messages, listeners want to reduce their comprehension effort, and therefore require explicitness and clarity. Thus, language reflects a compromise between these two conflicting production and comprehension pressures. The results of artificial language learning studies indicate that in the absence of a communication pressure the pressure for learnability trumps the pressure for usability in the evolution of language. When a communication pressure is present experimental-semiotic studies suggest that usability trumps learnability.

The results of experimental-semiotic studies are restricted to usability measures taken from those who created the communication system and do not extend to usability among those who will inherit the evolved system (i.e., a second generation). Using a paradigm similar to [27], the present study compares the ease with which the sign systems generated in small and larger populations can be learned (i.e., understood) and used (i.e., produced) by a second generation of naïve participants (using the corpus of signs collected by [21]). This distinction between comprehension and production processes is basic to psycholinguistic models of language processing [30].

We predict that the main pressure operating during the evolution of human communication systems is usability (i.e., production efficiency and fidelity). In the context of the present study, we predict that the Late evolved symbolic signs will be less learnable than the Initial iconic signs. However, we predict that this learning deficit will be offset by improved usability. Specifically, we predict that the Late evolved signs will exhibit three distinct production advantages (compared to the Initial iconic signs): they will be less cognitively demanding to bring to mind and plan, they will be more efficient to execute and they will be more faithfully reproduced.

A further set of predictions is made with regard to population size. Several scholars have argued that principles of Darwinian evolution can help explain the dynamics of cultural evolution [31]–[33]. In small 2-Person groups the initial communicative variants are subject to guided variation, causing them to become simpler across repeated interactions. In the larger 8-Person groups the signs are subject to guided variation plus a content bias. With considerable variation, a content bias can act on the pool of competing variants, selecting the form that best suits the communicative needs of the broader population. If correct, we predict that the learning deficit seen for the Late signs will be more muted for signs that evolve in a larger population (replicating [27]), and that any production benefit that is observed for the Late signs will be stronger for signs that evolve in a larger population.

## Method

The study reported received approval from the University of Western Australia Ethics Committee. All human participants viewed an information sheet before giving written consent to take part in the study. The information sheet and consent form were both approved by the Ethics Committee.

### Participants and Apparatus

One hundred and four psychology undergraduate students participated in exchange for partial course credit or payment. Participants were individually tested in sessions lasting forty-five minutes. Stimuli were presented and responses were recorded on a personal computer (using bespoke software created in PHP, JavaScript and MySQL).

### Materials and Design

Stimulus materials were drawn from Fay et al [21]. The stimuli consisted of 512 drawings produced in four 8-Person groups and 512 drawings produced in sixteen 2-Person groups. The first drawing of each concept was sampled from each interacting pair from an 8-Person group (i.e., *Initial* drawings; 16 concepts×16 pairs) when they were playing the Pictionary-like game with their first partner (Partner 1). The first drawing of each concept produced with their final partner (Partner 7) was also sampled (i.e. *Late* drawings; 16 concepts×16 pairs). Drawings of each concept were sampled from the same position from participants allocated to the 2-Person group condition (i.e., Game 1 and Game 37).

Each participant in the current experiment was presented with either the Initial or Late drawings (randomly allocated). Half of the drawings came from a pair from a 2-Person group and half from a pair from an 8-Person group. In total, each participant saw 32 signs (16×2-Person drawings and 16×8-Person drawings). A mixed design was used: 2-Person or 8-Person group was a within-participant factor and Initial or Late drawings were between.

### Procedure

The experiment took place in two phases: Phase 1 assessed learnability and Phase 2 assessed usability. In Phase 1, participants learned the identity of the drawings produced in the 2-Person and 8-Person group conditions (16 drawings per condition). Phase 1 learning trials were initiated by the presentation of a fixation cross in the middle of the computer screen (500 msec) followed by the presentation of the target drawing (1500 msec followed by a blank screen). Participants then tried to identify the referent of the drawing from a list of 20 concepts (16 targets and 4 distractors; see Table 1 for a complete listing). The drawings were presented in a random order and referent selection was made by mouse click. Participants were given corrective feedback: the correct answer was highlighted in green. To proceed to Phase 2, participants had to correctly identify the referent of each drawing three times in a row. When a drawing had been correctly identified three consecutive times it was removed from the pool of drawings being tested. When no drawings remained in the pool, Phase 1 was complete.

**Table 1:** The set of concepts communicated by participants in [21].

Places	People	Entertainment	objects	abstract
Art Gallery	Arnold Schwarzenegger	Cartoon	Computer Monitor	Homesick
Parliament	Brad Pitt	Drama	Microwave	Loud
Museum	*Keanu Reeves*	Soap Opera	*Toothbrush*	Poverty
Theatre	Russell Crowe		Television	*Bright*
*Hospital*				

Distractor items are given in italic.

In Phase 2 participants had to graphically reproduce the drawings learned in Phase 1. Phase 2 production trials were initiated with the presentation of a fixation cross in the middle of the computer screen (500 ms) followed by a text prompt that indicated the to-be-drawn concept (presented at the top of the screen and remained onscreen until the trial was completed). Drawings were produced on the onscreen digital whiteboard using the computer mouse. Participants were instructed to reproduce the associated drawing as accurately and as quickly as possible. Once completed, they clicked on a button at the bottom of the screen to bring the current trial to an end and proceed to the next trial. The computer recorded participants’ drawing latency (time, in msecs, between the presentation of the text prompt and drawing onset) and drawing execution time (time, in msecs, between drawing onset and trial completion) for each trial.

The 2-Person and 8-Person conditions were tested separately. That is, participants completed Phase 1 and 2 for each set of drawings sequentially. The order (2-Person, 8-Person) was counterbalanced across participants. To reduce any interference effects, participants completed a distractor task (copying unrelated drawings) between conditions.

## Results

A technical problem meant that the data for 26 participants was incompletely recorded; all data from these participants was excluded. This reduced our sample size to 78 (N for Initial signs = 48; N for Late signs = 30). Four sets of analyses are reported. The first examines the ease with which the second generation of participants was able to learn the communication systems generated in the small and larger populations. The remaining analyses examine the second generation’s ability to use the sign systems produced in the small and larger populations.

### Learning the Communication Systems Generated in Different Sized Populations

As people repeatedly interact the descriptions they use to describe the concepts become increasingly succinct and abstract. This pattern, observed in natural language studies [34], [35] and experimental-semiotic studies [15], [18], [21], makes communication more efficient. Although there is no comprehension cost to those actively involved in this process, non-active, passive learners have more difficulty picking out the referent of the later more symbolic descriptions compared to the earlier more iconic descriptions [15], [36]. This learnability loss is reduced when the descriptions are negotiated within a larger population [27].

**Figure 3:**
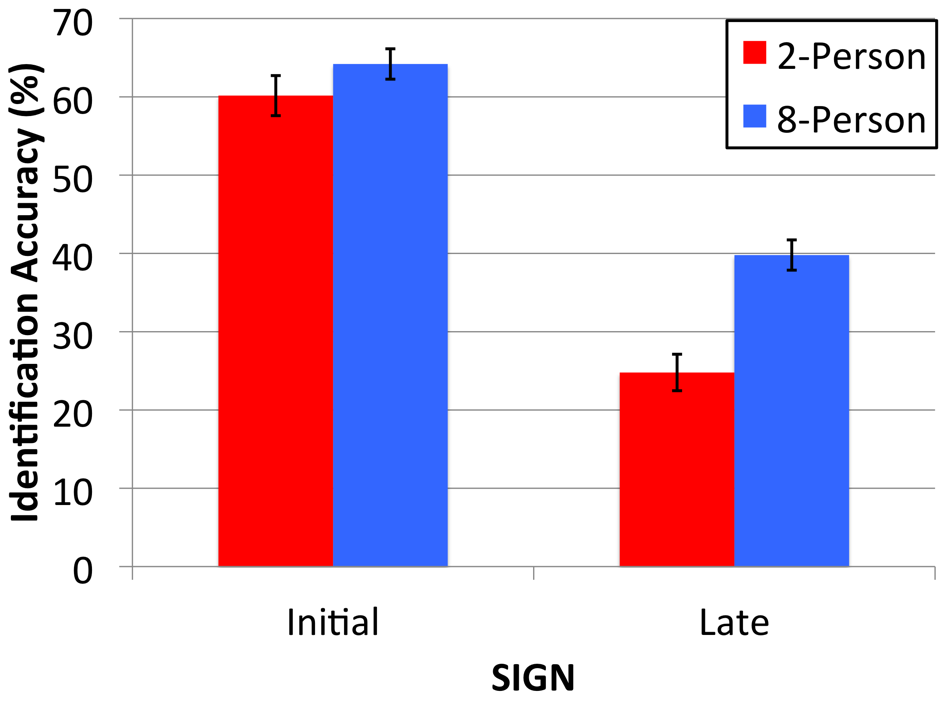
Mean identification accuracy (%) for Initial and Late 2-Person (red bars) and 8-Person (blue bars) signs. Error bars indicate the standard error of the means.

The first analysis seeks to replicate this finding by testing the ease with which a second generation of naïve participants is able to learn the meaning associated with the signs created in a 2- and 8-Person group. We tested participants’ ability to guess the referent of each sign on first encounter and the number of trials taken to learn each sign-meaning mapping to criterion (correct identification on three consecutive encounters). Figure 3 displays participants’ mean identification accuracy for the signs produced in the 2- and 8-Person groups on first encounter. Identification accuracy for the Initial signs is equivalent across the 2- and 8-Person conditions. For both conditions, participants’ identification rates were higher for the more visually complex Initial signs compared to the simpler Late signs. In addition, participants were better able to identify the referent of the Late 8-Person group signs when compared to the Late 2-Person group signs. This was confirmed by ANOVA.

Mean percentage accuracy scores were entered into a mixed design ANOVA that treated Group (2-Person, 8-Person) as a within-participant factor and Time (Initial, Late) as between. This returned main effects for Group [F(1,76) = 16.68, p<.001, η_p_^2^ = .18] and Time [F(1,76) = 156.24, p<.001, η_p_^2^ = .67], in addition to a Group by Time interaction [F(1,76) = 5.53, p = .02, η_p_^2^ = .07]. The interaction can be explained by the comparable accuracy of participants when identifying the referent of the Initial signs [t(47) = 1.29, p = .20], and the superior performance of the Late 8-Person signs over the Late 2-Person signs [t(29)>4.85, p<.001, d = 1.28]. The same pattern is returned when we analyze the number of trials it took participants to learn the different types of sign-meaning mappings to criterion (correct identification on three consecutive encounters). For both conditions the Initial sign-meaning mappings required fewer trials to learn to criterion compared to the Late signs. Initial sign-meaning mappings from each condition were learned at a similar rate (2-Person M = 3.61 trials, 8-Person M = 3.50 trials, t(47) = 1.62, p = .11). The Late 8-Person sign-meaning mappings were learned to criterion more quickly than Late 2-Person signs (2-Person M = 4.34 trials, 8-Person M = 3.88 trials, t(29) = 5.74, p<.001, d = 1.08).

In summary, naïve participants were able to guess the meaning of the Initial signs produced in the 2- and 8-Person groups equally well. Furthermore, the visually complex Initial signs were easier to understand that the simpler Late signs. While this might seem counter to an adaptive evolutionary account, it is counteracted by the usability benefits associated with the Late evolved signs (documented in the next section). Importantly, and despite their comparable visual complexity, the referent of the Late 8-Person group signs was more accurately identified on first encounter and quicker to learn to criterion when compared to the Late 2-Person signs. This finding replicates [27]. Like [27] we believe this learning benefit arises from the greater residual iconicity of the signs that evolve in larger populations.

### Using the Communication Systems Generated in Different Sized Populations

The current study’s key prediction is that human communication systems evolve to be usable. Three complementary usability measures were taken. Drawing latency - the time from display of a concept label to first pen down - is a cognitive measure, capturing the ease with which the participants can bring to mind the associated sign and plan its execution. Drawing execution time - from first pen down to last pen up - is a behavioral measure, capturing sign execution efficiency. Drawing fidelity measures the accuracy with which the acquired sign system is reproduced. The fidelity measure is particularly important, because, for a communication system to survive and for it to work people must use it in the same way (i.e., it must be shared). On account of the observed reduction in sign complexity through interaction, we predict that the Late evolved signs will exhibit improved usability on each measure (compared to the Initial signs). Furthermore, if larger populations generate better-adapted signs, this will be reflected by enhanced usability performance on these production measures.

**Figure 4:**
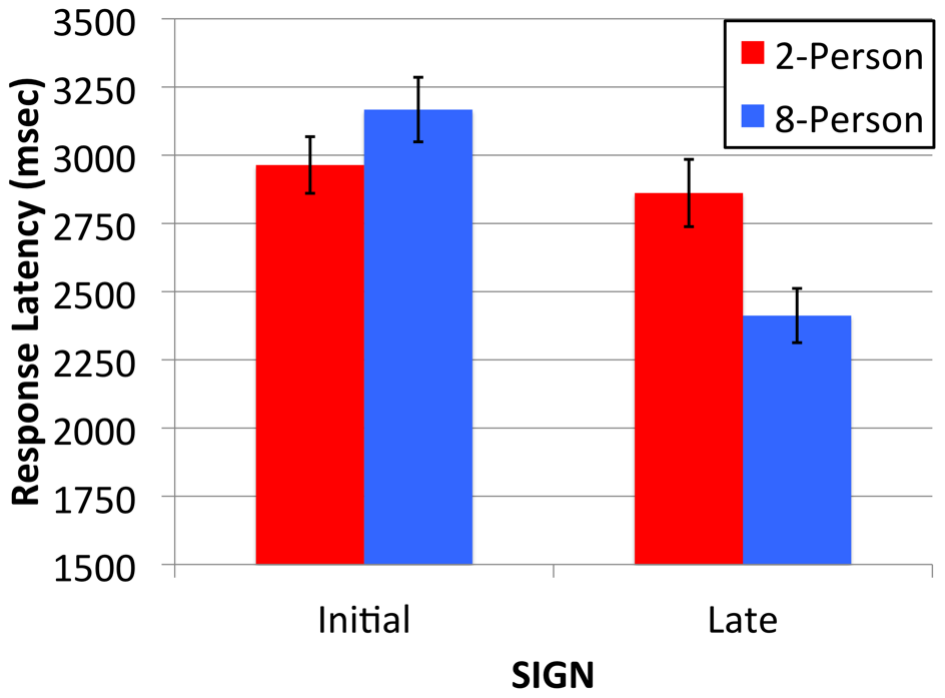
Mean response latency (msec) for Initial and Late 2-Person (red bars) and 8-Person (blue bars) signs. Error bars indicate the standard error of the means.

Figure 4 shows participants’ mean drawing latency (in msecs) for the different types of sign. The mean drawing latency scores are slightly shorter for the Initial 2-Person signs than for the Initial 8-Person signs. Being more visually complex, the Initial signs took longer to bring to mind and plan than the Late 2- and 8-Person signs. Importantly, and despite the comparable visual complexity of the Late signs, the signs that evolved in the larger 8-Person groups demonstrated a shorter drawing latency. These observations were confirmed by ANOVA.

Data were first screened for outliers following the method advocated by [37]. Extreme scores were replaced by the upper/lower bound. This accounted for 6.25% of the data. The same mixed-design ANOVA described earlier was used. This returned a non-significant main effect of Group [F(1,76) = 2.34, p = .14] and a statistically significant main effect of Time [F(1,76) = 8.80, p = .004, η_p_^2^ = .10]. As before, ANOVA returned a Group by Time interaction [F(1,76) = 15.71, p<.001, η_p_^2^ = .17]. This is explained by a faster drawing onset for the Initial 2-Person signs compared to the Initial 8-Person signs (by 203 msec on average, t(47) = 2.04, p = .05, d = 0.26), whereas participants showed a faster drawing onset for the Late 8-Person signs compared to the Late 2-Person signs (by 449 msec on average, t(29) = 3.35, p = .002, d = 0.73).

**Figure 5:**
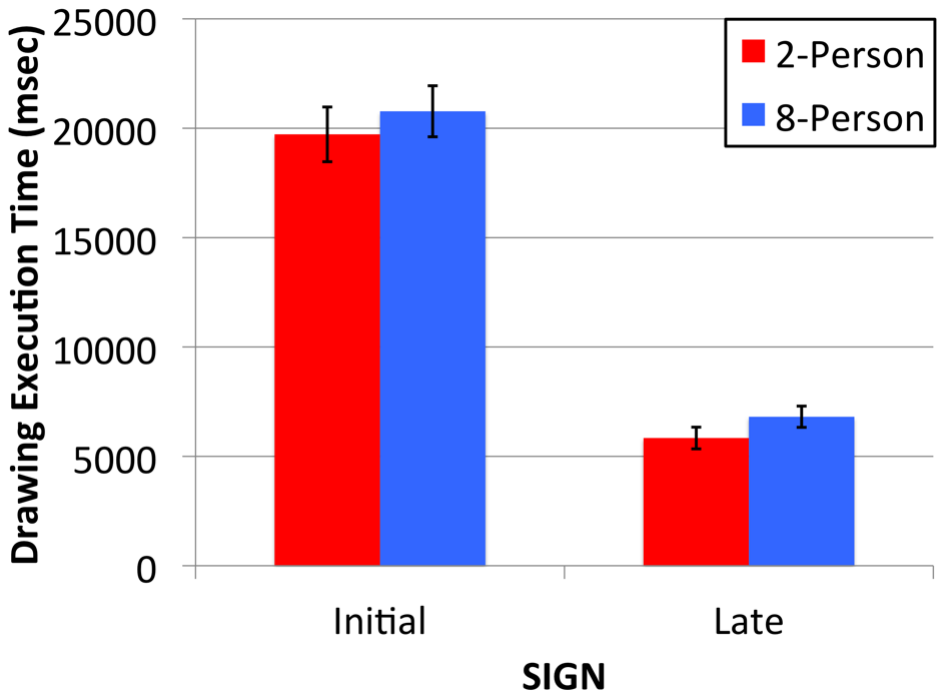
Mean time (msec) taken to produce Initial and Late 2-Person (red bars) and 8-Person (blue bars) signs. Error bars indicate the standard error of the means.

Figure 5 shows the time taken to execute the different types of sign. The time taken to execute the Initial and Late 2- and 8-Person signs did not differ. The visually simpler Late signs took much less time to execute compared to the more complex Initial signs. This is confirmed by ANOVA. Using the procedure described earlier, 3.13% of the data were identified as outliers and were replaced. ANOVA (same design as before) returned a main effect of Time [F(1,76) = 123.94, p<.001, η_p_^2^ = .62], indicating that the Late signs that evolved in the 2- and 8-Person groups were more rapidly produced than the Initial signs. No other effects reached statistical significance (Fs<1.09, ps>.30).

**Figure 6:**
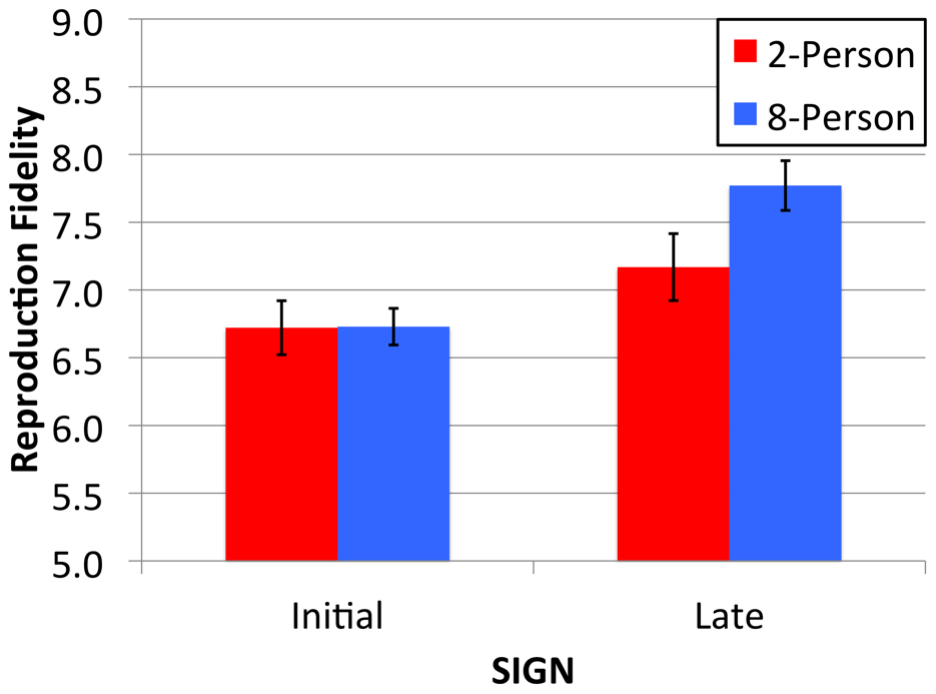
Mean reproduction fidelity (on a rating scale from 0–9, where 0 = very dissimilar and 9 = very similar) of Initial and Late 2-Person (red bars) and 8-Person (blue bars) signs. Error bars indicate the standard error of the means.

The final analysis examines the fidelity with which the different types of sign were reproduced. Pairs of signs (originals and reproduced signs) were coded for perceptual similarity by a rater naïve to the purpose of the study (on a scale from 0–9 where 0 = very dissimilar and 9 = very similar). A second rater (TME) then coded a subset of the pairs of signs (100). Correlating the similarity ratings across raters indicated strong inter-rater agreement [r(100) = .80, p<.001]. As Figure 6 shows, participants accurately reproduced the different types of signs; in each condition the similarity score between the original and reproduced sign is far greater than neutral similarity (i.e., a score of 4.5). Initial 2-Person and 8-Person signs were reproduced with similar fidelity. Sign reproduction fidelity increased from Initial to Late signs. In addition, the Late 8-Person signs were more accurately reproduced than the Late 2-Person signs. This was confirmed by ANOVA.

The drawing similarity scores were entered in the same mixed design ANOVA described earlier. This returned main effects of Group [F(1,76) = 4.53, p = .04, η_p_^2^ = .06] and Time [F(1,76) = 20.46, p = .002, η_p_^2^ = .12] in addition to a Group by Time interaction [F(1,76) = 4.30, p = .04, η_p_^2^ = .05]. The interaction effect can be explained by the comparable reproduction fidelity of Initial 2- and 8-Person signs [t(47) = 0.04, p = .97] and the higher reproduction fidelity of the Late 8-Person signs compared to the Late 2-Person signs [t(29) = 3.38, p = .002, d = 0.51]. Thus, despite their comparable visual complexity, participants more accurately reproduced the signs that evolved in the larger 8-Person groups.

In summary, naïve participants more efficiently and more accurately reproduced the simpler Late signs compared to the more complex Initial signs (both conditions). These usability findings are consistent with an adaptive evolutionary account. In addition, the signs that evolved in the larger populations exhibited distinct production benefits over the signs that evolved in the smaller populations: they were quicker to bring to mind and plan, and were more accurately reproduced. This occurred despite the signs that evolved in the 2- and 8-Person groups being of similar visual complexity.

## Discussion

A common theme shared by artificial language learning and experimental-semiotic approaches is that language evolution cannot be understood in terms of individual cognition and behavior. Instead, it must be understood as a collective phenomenon, a product of socio-cultural processes. However, the results of artificial language learning paradigms and experimental-semiotic approaches have been used to support different, but not necessarily mutually exclusive, cultural accounts of language. Artificial language learning studies argue for a top-down account; that individual-level cognitive biases guide language learning and language evolution [2], [38]. That is, as linguistic information is passed from person to person, or from generation to generation, it is modified in such a way that it increasingly reflects these cognitive biases, making the language easier for subsequent generations to learn. Experimental semiotic studies favor a bottom-up account, stressing the role social situational constraints to language evolution [21], [39]. On this account human communication systems are shaped and reshaped through the social interactions of its users to meet their emergent communication needs.

This present study examines the ease with which human communication systems are acquired by a second generation of users. More specifically, it asks if communication systems evolve to be easy to learn (as suggested by artificial language learning studies) or easy to use (as suggested by experimental-semiotic studies), and the effect that population size has on these competing acquisition pressures. Our results indicate that sign systems evolve to become more efficient to produce and more accurately reproduced, but also harder to comprehend. Although learnability and usability are both important acquisition pressures, our results suggest that the pressure for usability trumps the pressure for learnability in the evolution of human communication systems. In addition, communication systems that evolve in larger populations are easier for a second generation to learn and use compared to those that evolve in smaller populations.

Why do we see an opposing pattern of results across different experimental approaches to language evolution? Three reasons are discussed: intentionality, interaction and their focus on different aspects of language.

In the experimental language learning study discussed [7] participants were trained on an artificial language that they later tried to recall. What they recalled was passed to the next member of the transmission chain for learning and subsequent recall. Participants did not recall the language with the intention of it being understood by the next member of the transmission chain. Furthermore, they did not interact with the adjacent member of the transmission chain. Without communicative intent, and with no opportunity for interactive feedback, communication systems evolved that were easy to learn but had little functional value in terms of communication. In terms of the parity requirement for language, there was a push for ease of production with no pull for ease of comprehension. This led to a language that became easier to learn (it became easier to remember and recall due to a reduction in the number of unique object labels), but with reduced functional value in terms of communication (with no reduction in the number of objects, homonymy was increased). By contrast, participants engaged in experimental-semiotic studies have a clear communicative goal (communicate a range a pre-specified referents to a partner) and receive interactive feedback. With a push for ease of production and a pull for ease of comprehension, communication systems evolve among interacting agents that are efficient (i.e., easy to produce) and precise (i.e., easy to understand). This drive for communication efficiency is also observed in naturalistic studies showing that more frequently used words tend to be shorter [40]–[42]. What’s more, adding communicative intent and interaction to traditionally non-interactive transmission chain designs improves transmission fidelity [43] and usability [44].

It is important to reiterate that artificial language learning experiments and experimental-semiotic studies have explored different aspects of language evolution. Whereas artificial language learning studies focus on the individual-level cognitive biases that promote the emergence of linguistic structure, experimental-semiotic studies focus on the situational constraints that promote the evolution of language-like lexicons. Thus, the findings of experimental-semiotic studies can help us understand the rapid intra-generational evolution of the lexicon, and, once the lexicon reaches a particular size or threshold, artificial language learning experiments can help us understand the more gradual inter-generational evolution of linguistic structure [45]. Although intuitively appealing, this picture is complicated by an experimental-semiotic study [16] and an artificial language learning study [46] showing that efficient, precise and compositionally structured human communication systems can rapidly emerge through intra-generational social interaction. More research is needed to tease apart the interplay between the individual-level cognitive biases and situational constraints that shape the evolution of language.

The signs produced in 2- and 8-Person groups were incrementally simplified, making them more efficient to produce (cognitive effort to bring to mind and plan, and behavioral effort to execute) and more accurately reproduced by a subsequent generation of users. They also became more difficult to understand (compared to the Initial signs). Thus, an increase in short-term comprehension learning pain was rewarded with a long-term gain in terms of a communication system that is optimized for efficient production and sharedness (a pre-requisite for any useful communication system, and for cumulative cultural evolution [47]). Sign refinement can be explained through guided variation in the different sized populations (i.e., directed variant simplification through individual use [22]). Importantly, the decrease in learnability for Late signs (from the Initial signs) was more muted in the larger populations, and the production benefits were more pronounced (compared to the Late signs that evolved in the smaller populations). We argue that this occurs on account of the greater sign variation and competition in larger populations. If, as predicted, sign selection in larger populations is driven by a content bias (i.e., a particular sign is adopted on account of its favorable content [22]) then we would expect the selected signs to be easier to learn, produce and replicate. This is supported by our data. What is it then about the Late signs that evolve in larger populations that confer these advantages?

We propose that a content bias selects information-efficient signs (in the sense of Shannon’s source coding theorem [48]). That is, it selects signs that are maximally informative and minimally complex. In other words, it selects simple iconic signs. This helps naïve participants guess the sign’s meaning, and efficiently and accurately reproduce its form. A correlation between the reported comprehension and production measures would indicate an important role for sign iconicity in sign production. Evolved sign iconicity (collapsed across the Late signs produced in 2- and 8-Person populations, and operationalized as the mean number of trials-to-learn) correlates with each production measure: latency [r(77) = .29, p<.05], execution time [r(77) = −.36, p<.01] and reproduction fidelity [r(77) = −.38, p<.01]. These correlations link comprehension and production processes, and show that more iconic signs (those requiring fewer trials to learn) are quicker to bring to mind and plan, are more faithfully reproduced, but also require more time to execute. Although drawing execution times increase as iconicity increases, the more iconic signs that evolve in larger populations did not entail a significant execution time disadvantage (see Figure 5). This is because they are as simple as the signs that evolve in the smaller populations (see [21]). This indicates that a content bias selects signs that are more information-efficient in larger populations. Sign languages exhibit a similar pattern: while the signs have become less iconic over time, they continue to exhibit considerable iconicity [49], [50] and this helps later generations learn the system [51]. Spoken languages, in which iconicity is realized by less obvious mechanisms such as morphology, phonaesthemes and syntax, may well be affected by the same content bias [52]–[54].

The extent to which a content bias operates in a directed manner is unclear. Encountering several different partners may cause members of larger populations to preferentially choose more iconic signs that will cater to their broader audience. It is equally plausible that they behave in a non-directed manner and choose signs on the basis of their communication success. Empirical studies and computer simulations support a non-directed egocentric account [26], [55], [56].

### Conclusions

Communication systems must be learnable if they are to survive. But this is not the whole story (see also [57]). The present study shows that human communication systems also evolve to be usable. Dramatic sign simplification (between interacting agents) makes the signs easier to bring to mind and plan, quicker to execute and improves sign reproduction fidelity for the next generation of users. This simplification and abstraction process also makes the signs difficult to understand. Thus, usability trumps learnability. Population size affected learnability and usability: the loss in learnability was more muted in the sign systems that evolved in larger populations, and usability was enhanced. Guided variation can explain the improved usability of signs that evolve in small and large populations: directed variant simplification as a function of individual use. A content bias can explain the enhanced performance of the sign systems that evolve in larger populations: with more sign variation a content bias can operate to preferentially select signs that are better adapted for production and comprehension. The content bias selects information-efficient iconic signs that provide an optimal compromise between learnability and usability for the next generation of users.
